# Associative white matter tracts selectively predict sensorimotor learning

**DOI:** 10.1038/s42003-024-06420-1

**Published:** 2024-06-22

**Authors:** S. Vinci-Booher, D. J. McDonald, E. Berquist, F. Pestilli

**Affiliations:** 1 Department of Psychological and Brain Sciences, Program for Neuroscience, Indiana University, Bloomington, IN USA; 2 Department of Psychology and Human Development, Vanderbilt University, Nashville, TN USA; 3 Department of Statistics, University of British Columbia, Vancouver, BC Canada; 4 Department of Psychology, Center for Perceptual Systems, Center for Theoretical and Computational Neuroscience, Center for Aging Populations Sciences, Center for Learning and Memory, University of Texas at Austin, Austin, TX USA

**Keywords:** Language, Neural circuits, Perception

## Abstract

Human learning varies greatly among individuals and is related to the microstructure of major white matter tracts in several learning domains, yet the impact of the existing microstructure of white matter tracts on future learning outcomes remains unclear. We employed a machine-learning model selection framework to evaluate whether existing microstructure might predict individual differences in learning a sensorimotor task, and further, if the mapping between tract microstructure and learning was selective for learning outcomes. We used diffusion tractography to measure the mean fractional anisotropy (FA) of white matter tracts in 60 adult participants who then practiced drawing a set of 40 unfamiliar symbols repeatedly using a digital writing tablet. We measured drawing learning as the slope of draw duration over the practice session and measured visual recognition learning for the symbols using an old/new 2-AFC task. Results demonstrated that tract microstructure selectively predicted learning outcomes, with left hemisphere pArc and SLF3 tracts predicting drawing learning and the left hemisphere MDLFspl predicting visual recognition learning. These results were replicated using repeat, held-out data and supported with complementary analyses. Results suggest that individual differences in the microstructure of human white matter tracts may be selectively related to future learning outcomes.

## Abstract (web summary)

A diffusion imaging study suggests that individual differences in learning may be selectively predicted by tissue properties of major white matter tracts in the brain. In this study, the left pArc and SLF3 tracts predicted drawing learning in adults.

## Introduction

Human learning is a complex phenomenon that varies greatly among individuals^1^. Individual differences in learning may be related to individual differences in the structural architecture of the brain^2–16^. The structural architecture of the brain contains large bundles of myelinated fibers called white matter tracts that carry communications among cortical regions. The connectivity of these white matter tracts dictates which cortical regions directly or indirectly communicate. The microstructure of these white matter tracts–their cellular tissue properties–are related to other communication parameters, for example, the speed of communication among cortical regions. Here, we tested the possibility that individual variability in the microstructure of major white matter tracts could predict individual variability in learning. We tested the selectivity of the mapping between tracts and learning outcomes by evaluating the relationship between the microstructure of major white matter tracts and individual differences in learning to draw and visually recognize previously unknown symbols.

Drawing is a sensorimotor learning experience that leads to at least two measurable learning outcomes: drawing learning and visual recognition learning. First, drawing practice increases the ability to perform the drawing task itself. As adults practice drawing forms, such as objects, shapes, or symbols, the drawings produced become increasingly recognizable^17^. In young children who are just learning to write letters of the alphabet, writing a letter of the alphabet becomes easier and faster^18,19^ and their productions become more legible with practice^20^. Second, drawing practice leads to changes in visual processing and memory for the symbols produced, effects that can occur implicitly without direct training^21–26^. For example, practice with drawing common objects increases visual recognition of those objects^21^ and practice writing pseudo-letters from a novel alphabet increases visual recognition for the practiced pseudo-letters^26^. As an individual practices repeatedly drawing a form, they not only become better at drawing that form but also become better at visually recognizing that form.

**Fig. 1:**
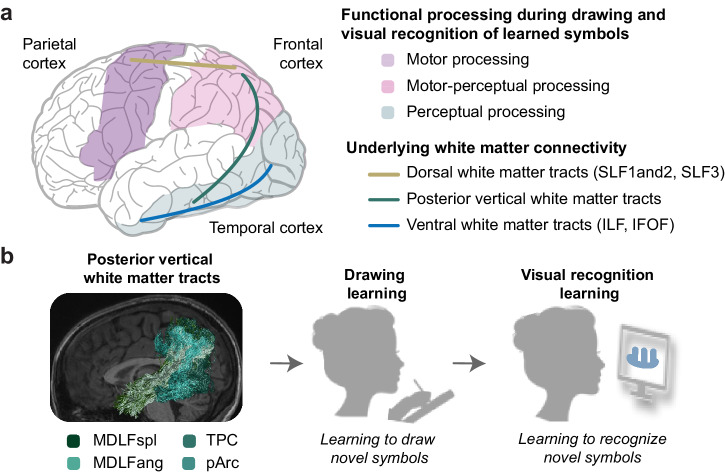
Background. **a** Major white matter tracts connect motor-oriented and perceptual-oriented cortical regions that coactivate during drawing and visual recognition of symbols. Connections allow for direct and indirect communication pathways between coactivated cortical processing regions. **b** The posterior vertical pathway (PVP) includes at least four major white matter tracts that allow for direct communication between motor-oriented parietal cortex and perceptual-oriented temporal cortex. SLF superior longitudinal fasciculus, separable into SLF1and2 and SLF3, ILF inferior longitudinal fasciculus, IFOF inferior fronto-occipital fasciculus, MDLFspl middle longitudinal fasciculus connection to the superior parietal lobe, MDLFang middle longitudinal fasciculus connection to the angular gyrus, TPC temporal to parietal connection; pArc posterior arcuate fasciculus.

A network of major white matter tracts connects cortical processing regions that co-activate during drawing and visual recognition of learned symbols (Fig. 1a)^27–35^. Within the dorsal motor cortex, the superior longitudinal fasciculus (SLF, separable into SLF1 and 2 and SLF3) directly connects frontal and parietal cortices^36^. Within the ventral perceptual cortex, the inferior longitudinal fasciculus (ILF) directly connects occipital and temporal cortices^37,38^ while the inferior fronto-occipital fasciculus (IFOF) connects occipital and prefrontal cortices^37,39^. Between the dorsal motor and ventral perceptual cortices, the long segment of arcuate fasciculus (Arc) and tracts within the posterior vertical pathway (PVP) directly connect the temporal cortex with dorsal motor-oriented cortical regions. The Arc directly connects temporal and frontal cortices^40,41^ while tracts within the PVP directly connect temporal and parietal cortices^42^. The PVP may be best thought of as a collection of tracts, including the posterior arcuate (pArc), the temporal-parietal connection (TPC), the middle longitudinal fasciculus connection to the angular gyrus (MDLFang), and the middle longitudinal fasciculus connection to the superior parietal lobe (MDLFspl) (Fig. 1b). When an individual draws or visually processes learned symbols, these major tracts allow for direct and indirect communications among coactivated brain regions, suggesting that the microstructure of these tracts may be related to the effectiveness or ineffectiveness of neural communications for learning.

The relationship between white matter and learning has been investigated using at least three broad approaches. The first is a cross-sectional approach that evaluates the relationship between current white matter and current behavioral abilities. Current white matter is indicative of white matter alterations that occurred in response to prior experiences and, similarly, current behavioral abilities are indicative of behavioral learning that occurred in the past^43^). A vast number of investigations have demonstrated correlations between white matter and behavior using this cross-sectional approach (for review^44,45^:. The second approach requires repeated sampling and evaluates the relationship between current white matter and future learning outcomes. Again, current white matter is considered indicative of alterations that occurred in response to prior experiences, but this second approach explicitly measures learning outcomes, often using an intervention or training protocol, rather than relying on current behavioral abilities to index prior learning. Results from this approach have demonstrated that white matter connectivity predicts learning sensorimotor tasks, such as learning to play novel piano sequences^6,8^, and that the microstructure of major white matter tracts can be used to predict future learning in multiple domains, such as semantic learning^10,11^, foreign language learning^12,46^, auditory learning^4,47^, visuomotor adaptation^7^, and face-name learning^9^. The third approach also requires repeated sampling but explicitly measures both white matter and learning outcomes over time, often by evaluating changes in white matter in response to an intervention or training protocol. Results from this approach have demonstrated that microstructural alterations can occur rather quickly in some areas, such as the hippocampus^48^, but require long and/or intensive training paradigms to effect measurable changes in the microstructure of major white matter tracts^49–52^. Although this third intervention approach is arguably the gold standard, the second approach that uses existing white matter to predict learning is advantageous for investigating major white matter tracts because it explicitly measures learning outcomes while avoiding long and/or intensive training paradigms.

Here, we evaluated the relationship between current white matter and future learning outcomes. More specifically, we investigated the relationship between the current microstructure of major white matter tracts and individual differences in performance on two learning outcomes following a single sensorimotor training task (Fig. 1). The sensorimotor training task was learning to draw unfamiliar symbols and the two learning outcomes measured were drawing and visual recognition learning. To test the possibility that the microstructure of major white matter tracts could selectively predict individual variability in learning (i.e., tract-selectivity), we employed a machine learning and model selection framework to select the group of tracts whose microstructure was most predictive of drawing learning and, separately, visual recognition learning. Measuring two learning outcomes allowed us to test the selectivity of white matter tracts to different learning outcomes (i.e., task-selectivity). Prior studies testing the relationship between tract microstructure and future learning outcomes have focused on tracts of interest (investigating only one or a few tracts) and have often tested a single learning outcome (investigating a single task without testing transfer of learning across behaviors). Furthermore, prior studies investigating the relationship between white matter and learning have rarely investigated the PVP tracts, excepting cross-sectional studies that have demonstrated a relationship between the microstructure of the pArc and drawing ability^53^ and reading ability^52,54–59^, suggesting that the PVP white matter tracts may be predictive of individual variability in drawing and visual recognition learning.

## Results

**Fig. 2:**
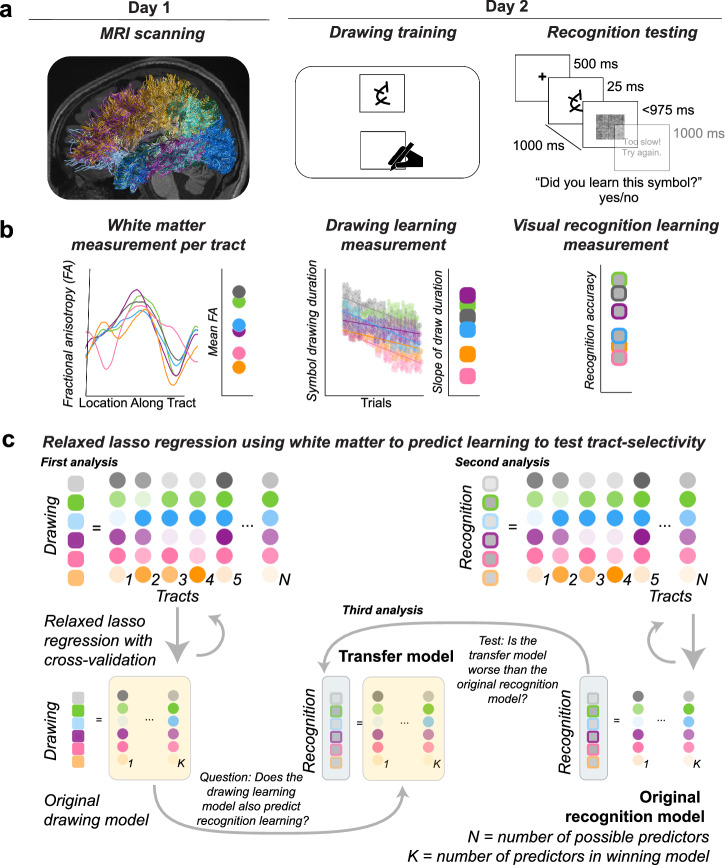
Experimental procedure, measurements, and modeling approach. **a** Overall procedure. All participants completed an MRI session before completing a session of drawing training and recognition testing. On Day 1, diffusion MRI data were collected in order to perform diffusion tractography and estimate tissue microstructure. On Day 2, participants completed 30 min of drawing training, including 40 symbols each drawn 10 times in random order (drawing training) followed by an old/new 2-AFC visual recognition test (recognition testing). **b** Calculations of white matter and learning measurements. First, a measurement of mean fractional anisotropy (FA) was obtained for each tract using a tractprofiles approach and averaging across the tract profile. Second, a measurement of drawing learning was obtained by estimating the linear slope of draw duration over trials. Third, a measurement of visual recognition learning was obtained by estimating the proportion of correct responses on the visual recognition test. The white matter measurements on day 1 were then used to predict the measurements of drawing learning and visual recognition learning on day 2. **c** Modeling approach. A relaxed lasso regression was used to identify the group of tracts that was most predictive of individual differences in drawing learning and, separately, most predictive of visual recognition learning. After predictors were selected for drawing learning and for visual recognition learning separately, we tested to see if the drawing learning model transferred to visual recognition learning (Transfer model). We found that the Transfer model was worse at predicting visual recognition learning than the model selected for visual recognition learning (Original model), demonstrating task-selectivity because the model selected for learning to draw symbols did not also predict learning to visual recognize symbols. Colors in **b** and **c** represent an individual participant; not all participants are depicted to save space.

We investigated the mapping between the microstructure of white matter tracts and two learning outcomes that arose from the same training experience to test both tract- and task-selectivity. We measured white matter microstructure in 60 adult participants who later completed a sensorimotor training task that required them to practice drawing a set of 40 unfamiliar symbols repeatedly using a digital writing tablet. After the training, participants were tested on their ability to visually recognize the 40 symbols that were previously unknown to them (Fig. 2a). We measured current white matter microstructure by averaging fractional anisotropy (FA) across each tract to produce one measure of FA for each tract and each participant at the start of the experiment. We estimated drawing learning by measuring the draw duration for each symbol drawing trial and calculating the slope of draw duration over the practice session. We estimated visual recognition learning by measuring accuracy in an old/new 2-alternative forced-choice (2-AFC) visual recognition task (Fig. 2b). Accuracy was selected over reaction time because both metrics demonstrated learning and accuracy captured more individual variability than reaction time (see Supplementary Information).

We employed relaxed-lasso (RL) regression methods^60,61^ to investigate the hypothesis that the microstructure of major white matter tracts could predict learning (in cross-validation terms) and, more specifically, that we would be able to identify a subset of tracts that together would be more predictive of learning than other tracts, i.e., tract-selectivity (Fig. 2c). The RL method tests a large space of potential models with different parameters (e.g., different combinations of tracts) and quantitatively selects the unique combination of parameters that explain the most variance in the dependent variable (i.e., learning outcome). Critically, RL does not guarantee identification of a small subset of tracts and is capable of selecting all tracts, if that provides the best fitting model^61^. We performed two separate RL regressions to identify the group of tracts that best predicted drawing learning and to identify a group of tracts that best predicted visual recognition learning. We directly compared the models selected for drawing learning and visual recognition learning to investigate the hypothesis that tract microstructure would be selectively related to learning outcomes, i.e., task-selectivity. We evaluated how well the model selected to predict drawing learning from tract microstructure would transfer to predicting visual recognition learning.

We directly tested a set of 22 white matter tracts that connect cortical regions known to support motor and sensory processing during symbol drawing^27–30,32,33,35,62,63^ (Fig. 1a): the first and second segment of the Superior Longitudinal Fasciculus combined (SLF1and2), the third segment of the Superior Longitudinal Fasciculus (SLF3)^36^, the Inferior Longitudinal Fasciculus (ILF)^37,38^, the Inferior Fronto-Occipital Fasciculus (IFOF)^37,39,64^, the arcuate fasciculus (Arc)^40,41^, and the PVP tracts that included the Posterior Arcuate (pArc), the Temporal-to-Parietal Connection to the Superior Parietal Lobe (TPC), the Middle Longitudinal Fasciculus Connection to the Angular Gyrus (MDLFang), and the Middle Longitudinal Fasciculus Connection to the Superior Parietal Lobe (MDLFspl)^42^. We also included two additional vertical tracts, the Vertical-Occipital Fasciculus (VOF) in the posterior cortex and the Frontal Aslant Tract (FAT) in the anterior cortex. The left and right hemispheres were kept separate for each of these 11 tracts, for a total of 22 white matter tracts tested. All analyses were also conducted with a more extended set of tracts using the same approach, and results were consistent with the theory-driven selection of 22 tracts that we report in the main text. We report the results of the analyses with a more extended set of tracts in Supplementary Table 1.

We tested our hypothesis that a select group of white matter tracts would predict learning in three separate analysis steps (Fig. 2c). In the first analysis, we specified an initial model that we called the original drawing learning model using an RL regression to select a set of white matter tracts that explained the most variance in drawing learning. In the second analysis, we constructed a second model that we called the original recognition learning model using a second RL regression to select tracts that explained the most variance in visual recognition learning. In the third analysis, we evaluated whether the relationship between pre-training white matter microstructure and drawing learning found in the first analysis would translate to a second learning outcome, namely visual recognition learning. We report a complementary analysis using simple (marginal) linear regressions in to evaluate the ability of the microstructure of any tract to independently predict each learning outcome, reporting findings that are consistent with the results of the RL analyses reported in the main text (see Supplemental Figs. 1 and 2 and Supplemental Table 2 and 3).

### First analysis

#### Predicting drawing learning from the microstructure of major white matter tracts

We used an RL regression and model-selection via cross-validation to identify the subset of white matter tracts that best predicted sensorimotor learning, i.e., drawing learning. We hypothesized that the microstructure of a select group of white matter tracts prior to learning would predict individual differences in learning to draw unfamiliar symbols.

**Fig. 3:**
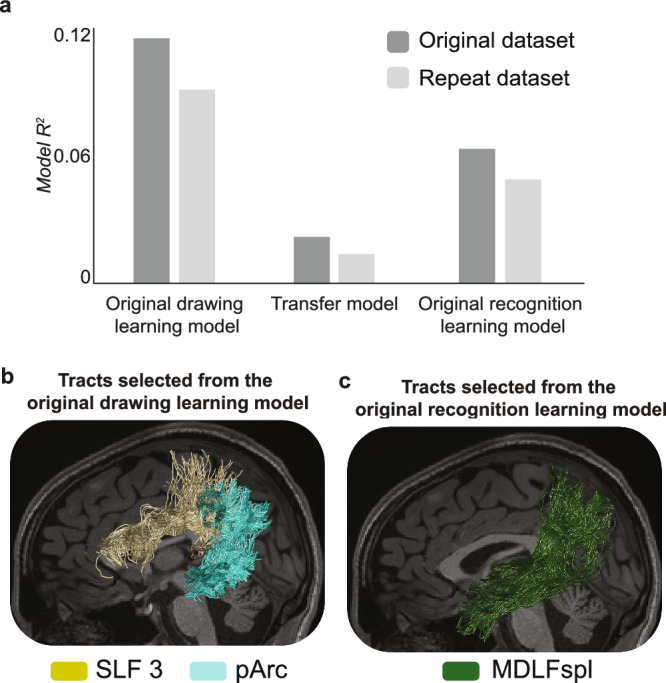
Associative white matter tracts selectively predict learning. **a** Relaxed lasso (RL) regressions were performed to select the group of tracts that best predicted drawing learning and visual recognition learning separately. The bar plot depicts the OLS *R*^2^ values for the model selected to predict drawing learning (left) and the model selected to predict visual recognition learning model (right). The bar plot also depicts the OLS *R*^2^ values for a transfer model that was constructed to test if the model selected for drawing learning might also predict visual recognition learning. Relative to the original drawing learning model and the original visual recognition learning model, the transfer model was very poor. A Cox test and a J-test both confirmed that the model selected for drawing learning did not transfer to predicting a second learning outcome, i.e., visual recognition learning. **b** Tracts selected to predict drawing learning, included the left SLF3 and the left pArc. Tracts from one representative participant are displayed. **c** Tracts selected to predict visual recognition learning included the left MDLF-spl in the original and repeat dataset. Tracts from one representative participant are displayed. We note that the model *R*^*2*^ values depicted in the plot are consistent with the results reported in Table 1; therefore, the *R*^2^ value for the original dataset includes both the left MDLFspl and the left temporal-to-parietal connection to the superior parietal lobe (TPC) while the *R*^*2*^ value for the repeat dataset includes only the left MDLFspl. SLF 3: third segment of the superior longitudinal fasciculus, pArc posterior arcuate, MDLFspl middle longitudinal fasciculus connection to the superior parietal lobe.

Results of the RL analysis supported our hypothesis: the microstructure of a select group of white matter tracts prior to learning predicted drawing learning. The RL regression optimized to predict drawing learning revealed that two left hemisphere tracts explained the most variance in drawing learning: the left pArc and left SLF3 (Table 1; Fig. 3). These two tracts were selected from 22 potential tracts and from multiple competing models^61^, suggesting tract-selectivity. With drawing learning as the dependent variable, the winning model included only the left pArc and the left SLF3, with an OLS *R*^*2*^ = 0.1180 for the final selected model. The relationships between the microstructure of the left pArc and left SLF3 were positive, such that participants with higher FA in those tracts were participants who were the quickest at learning to draw the unfamiliar symbols.

**Table 1:** Models selected for each learning outcome using relaxed lasso regression

Response Variable	Predictor	*β*	*S.E*.	*R*^*2*^
*Drawing learning*	Left pArc	0.2118	0.2654	0.1180
	Left SLF3	0.1772	0.2600	—
*Drawing learning (repeat dataset)*	Left pArc	0.1968	0.2517	0.0969
	Left SLF3	0.1448	0.2569	—
*Visual recognition learning*	Left MDLFspl	-0.6290	1.4424	0.0662
	Left TPC	-0.4025	1.1971	—
Visual recognition learning (repeat dataset)	Left MDLFspl	-0.7562	1.4981	0.0416

Summary statistics are estimated via OLS using the final model predictors.

This result was replicated in a held-out, repeat dataset where the same analysis identified a winning model that, again, included only left pArc and the left SLF3, with an OLS *R*^*2*^ = 0.0969. We found similar results in a complementary series of marginal linear regressions that, again, suggested a relationship between the microstructure of the left pArc and the left SLF3 and individual differences in drawing learning (see Supplemental Figs. 1 and 2 and Supplemental Table 2 and 3).

### Second analysis

#### Predicting visual recognition learning from the microstructure of major white matter tracts

Drawing training also results in changes in visual recognition for the practiced symbols that can occur during symbol drawing training even in the absence of directly training visual recognition for the symbols^26^. We, therefore, performed a second RL regression and model-selection via cross-validation procedure to identify the subset of white matter tracts that best predicted visual recognition learning (Fig. 2b). Similar to the first analysis, we hypothesized that the microstructure of a select group of tracts prior to learning would predict individual differences in visual recognition learning for the practiced symbols.

An RL model was specified that predicted visual recognition learning (accuracy) given the 22 predictors previously used to select the model for drawing learning. Results of the RL analysis supported our hypothesis: a select group of tracts prior to learning predicted individual differences in visual recognition learning. More specifically, two left hemisphere tracts were selected to predict visual recognition learning: left MDLFspl and left TPC, with an OLS *R*^*2*^ = 0.0662 (Table 1; Fig. 3). The relationship was negative, such that participants with higher FA in the left MDLFspl and left TPC were participants who demonstrated the lowest visual recognition learning.

This result was partially replicated in the held-out dataset; the same analysis applied to a repeat dataset identified only the left MDLFspl, with an OLS *R*^*2*^ = 0.0416. The only tract identified for visual recognition learning in both the original and repeat datasets was the left MDLFspl; however, this tract was not significantly related to visual recognition in the complementary simple (marginal) linear regressions (see Supplemental Figs. 1 and 2 and Supplemental Table 2 and 3). The results of the marginal linear regressions did not reveal a significant relationship between the microstructure of the left MDLFspl or any other individual tract and visual recognition learning.

### Third analysis

#### Directly testing the task-selectivity of major white matter tracts for drawing learning

Our third analysis tested task-selectivity by evaluating if the model selected for drawing learning could also predict a different learning outcome, namely visual recognition learning. Drawing and visual recognition learning were two different learning outcomes that occurred during the same sensorimotor training session and, therefore, make for a strong test of selectivity to learning outcomes. We hypothesized that the relationship between white matter and individual differences in drawing learning would not transfer to individual differences in visual recognition learning, that tracts predictive of drawing learning would demonstrate a selectivity for drawing relative to visual recognition.

To evaluate if the model selected from drawing learning could transfer to visual recognition learning, we evaluated if the predictors selected for drawing learning could explain any variance in visual recognition learning beyond the variance explained by the original predictors originally selected for visual recognition learning using a Cox test for non-nested models to (Fig. 2c)^65^. The Cox test assesses if any variance in an original dependent variable that is not explained by the original set of predictors could be explained by a different set of predictors. This is the most appropriate approach; directly comparing the predictors selected for drawing learning and the predictors selected for visual recognition learning would be circular because the RL regression already demonstrated that the predictors selected for visual-recognition learning produce better fits for visual recognition learning than the other potential predictors, including the predictors selected for drawing learning. Rather, the most appropriate test is to test if the predictors selected for drawing learning could explain any additional variance in visual recognition learning that is not already explained by the predictors selected for visual recognition learning.

The Cox test was performed in two steps. First, we obtained the fitted values from the original visual recognition model by regressing the original dependent variable, visual recognition learning in this case, on the original predictors identified for visual recognition learning (i.e., the left MDLFspl and left TPC). Second, we constructed a transfer model by regressing the fitted values from the original visual recognition model on the predictors originally selected for drawing learning (i.e., the left pArc and left SLF3). This second step evaluates if the predictors selected for drawing learning could explain any variance in visual recognition learning that was not explained by the original visual recognition model. If the fit of the transfer model can explain variance remaining from the original visual recognition model, such a result would suggest that the tracts selected as most predictive of drawing learning might transfer to visual recognition learning and are, therefore, not likely specific for drawing. On the other hand, if the transfer model is unable to explain additional variance, such a result would suggest that the drawing learning predictors do not transfer to visual recognition learning. We also performed a Cox test to evaluate if the predictors selected for visual recognition learning might transfer to drawing learning using the same analysis approach.

Results suggested that the relationship between white matter and drawing learning did not transfer to visual recognition learning, and vice versa (Fig. 3). The Cox-test demonstrated that the predictors originally selected for drawing learning (i.e., with predictors corresponding to the left pArc and left SLF3) were not able to explain additional variance in visual recognition learning beyond the variance explained by the original recognition model (i.e., with predictors corresponding to the left MDLFspl and the left TPC), *z* = 0.1382, *p* = 0.890, and that the predictors originally selected for visual recognition learning were not able to explain additional variance in drawing learning beyond the variance explained by the original drawing learning model, *z* = -3.3122, *p* = 0.0009. We performed a complementary J-test that produced results consistent with the results of the Cox-test reported here (see Supplementary Information). In sum, results suggest that predictors originally selected for one learning outcome were not transferable to a second learning outcome, indicating some selectivity between tracts and learning outcomes.

These results were replicated in a held-out repeat dataset; the same analyses suggested that the predictors originally selected for drawing learning were unable to explain additional variance in visual recognition, *z* = 5.4093, *p* = 6.3 × 10^-8^, and that the predictors originally selected for visual recognition learning were unable to explain additional variance in drawing learning, *z* = 0.2548, *p* = 0.7988. Results of the J-test were again consistent with results of the Cox test (see Supplementary Information).

## Discussion

The current work employed a machine-learning model selection approach to demonstrate selectivity of the mapping between the existing microstructure of major white matter tracts and future learning outcomes. We used diffusion measurements of white matter tissue to predict individual differences in two learning outcomes that arose from a single sensorimotor training task. The sensorimotor training task consisted of drawing symbols that were previously unknown, and the two learning outcomes included learning to draw the novel symbols and learning to visually recognize those symbols. Results suggested that two left hemisphere white matter tracts, the left pArc and the left SLF3, selectively predicted individual differences in learning to draw unfamiliar symbols but not learning to visually recognize those same symbols. The relationship between the pre-training microstructure of these two left hemisphere tracts and drawing learning was found using two independent datasets and two separate statistical analyses (see Results and Supplementary Information). On the other hand, the relationship between pre-training microstructure and visual recognition learning varied marginally depending on the dataset and statistical analysis but suggested that the pre-training microstructure of the left MDLFspl may be related to visual recognition learning. Overall, results suggest that individual differences in the microstructure of human white matter tracts may be selectively related to learning outcomes, even when those outcomes arise from a single experience.

The current work is the first, to our knowledge, to demonstrate a selective mapping between major white matter tracts and human learning. Few studies have tested the mapping between white matter tracts and multiple learning outcomes, leaving the current literature unable to conclude that some tracts are more related to learning than other tasks (tract-selectivity) and more related to learning one task than other tasks (task-selectivity)^4,6–12^. Our results add to prior human work that observed widespread changes across multiple white matter tracts during an intensive intervention^52^. Although interventions often target one learning outcome (e.g., reading), they often impart learning in other domains that are not directly targeted (e.g., attention, social interactions). The onset of an intensive intervention might promote widespread changes^52^ and our results suggest that only a few of those changes are related to the targeted learning outcome. By using tract microstructure to predict future learning and assessing more than one learning outcome, our results demonstrate a selective mapping between white matter tracts and learning outcomes that likely emerges over time periods much longer than is typical intervention timelines.

Two independent analyses demonstrated that the microstructure of two left hemisphere white matter tracts selectively predicted drawing learning: the left pArc and the left SLF3. The first analysis tested tract-selectivity using a relaxed lasso regression to identify a group of white matter tracts that, together, predicted drawing learning from a set of 22 potential tracts. Each tract entered into the relaxed lasso (RL) regression was selected based on its unique anatomical connectivity in relation to the functional responses observed during drawing in prior works^27–30,35^, including SLF 1 and 2 (combined), SLF3, pArc, TPC, MDLFspl, MDLFang, Arc, ILF, IFOF, VOF, and FAT in the left and right hemispheres. The RL analysis indicated that the left SLF 3 in dorsal cortex and the left pArc in the PVP comprised the group of tracts that explained the most variance in drawing learning. The second analysis tested the task-selectivity of the drawing learning model (i.e., the left pArc and left SLF3) for drawing by evaluating if the predictors selected for drawing learning might transfer to a second learning outcome, visual recognition learning. Results revealed that the drawing learning model did not transfer to visual recognition learning, suggesting a degree of task-selectivity between the microstructure of the left pArc and left SLF3 and drawing learning. Furthermore, results from both analyses were replicated in a held-out repeat dataset, providing strong evidence that individual differences in the microstructure of the left pArc and left SLF3 are selectively related to individual differences in learning to draw unfamiliar symbols.

Both white matter tracts selected to predict drawing learning were in the left hemisphere, suggesting that learning to draw unfamiliar symbols might be supported by left-lateralized communications conveyed along the pArc and SLF3, consistent with evidence of left-lateralization of functional processes during drawing. Literate adults engage a left-lateralized cortical system during drawing, including regions within the frontal motor, parietal, and ventral temporal lobes^27–35^, and these regions are joined by the left pArc and left SLF3^36,40,42^. Furthermore, work in children has demonstrated that the pArc is correlated with individual differences in drawing ability in the left but not the right hemispheres, even after controlling for age^53^. We and others^66–68^ have suggested that the left-lateralization of white matter supporting drawing learning may be related to the left-lateralization of language processing. In the current study, participants drew symbols that resembled letters of the Roman alphabet and might have relied on the white matter architecture that had been optimized for drawing through extensive life experiences with handwriting letters of the alphabet, an unmistakably language-oriented task. Comparing tract-selectivity in literate and non-literate adults might provide further support for the role of life experiences in individual differences in the microstructure of major white matter tracts that support cortical communications during drawing learning.

The arcuate fasciculus (Arc) is often segmented into three white matter tracts that allow for at least two different communication pathways, including a long segment (connecting temporal and frontal cortices), an anterior indirect segment (connecting parietal and frontal cortices), and a posterior segment (connecting parietal and temporal cortices)^40,41^. This segmentation is of great interest to language and reading research because it allows for at least two neural communication pathways during language-oriented tasks: the direct and indirect pathways. The direct pathway is the long segment because it directly connects processing for language perception, such as graphemes (i.e., visual symbols), thought to occur in the temporal cortex with motor processing for language production, such as pronouncing phonemes (i.e., symbol sounds) or writing graphemes (i.e., symbol writing) thought to occur in the frontal motor cortex^40^. The indirect pathway accomplishes the same connection between perceptual and motor processing but does so indirectly by passing through the parietal cortex. In our segmentation approach, the direct pathway is captured by the left Arc and the indirect pathway is captured by the left pArc and left SLF3^40–42,69,70^. The SLF3 is essentially one and the same with the anterior indirect segment of the Arc^69–71^ and the pArc is essentially one and the same with the posterior segment of the Arc^42,71^. Therefore, our results demonstrate that the indirect pathway is predictive of drawing learning in adults suggesting that parietal involvement might be especially important for drawing learning because we found that the indirect pathway (left pArc, left SLF3) selectively predicted drawing learning but not the direct pathway (left Arc).

The current work suggests that segmenting the PVP into four white matter tracts may reveal unique relationships with behavior and cortical functioning. The PVP can be segmented into four white matter tracts, however the utility of segmenting the PVP into these four white matter tracts has not yet been determined. For example, one study reported no difference in the developmental trajectory of the microstructure of the PVP tracts^53^. Our results demonstrate that the pArc and MDLFspl selectively predicted different learning outcomes that arose from the same training task. While the pArc predicted drawing learning, the MDLFspl predicted visual recognition learning. The pArc connects the posterior ventral-temporal cortex with the inferior parietal lobe (IPL) where processing is largely associated with visually-guided actions with the hands^72^; the MDLFspl connects the anterior ventral-temporal cortex with the superior parietal lobe (SPL) where processing is largely associated with visual attention^73^. Thus, the pArc may predict drawing learning by supporting communication between perceptual processing in posterior ventral-temporal cortex and visual processing for hand actions in the IPL while the MDLFspl may predict visual recognition learning by supporting communication between perceptual processing in anterior ventral-temporal cortex and visual attention in the SPL. Future work will be necessary to continue investigating the mapping between the four PVP tracts and learning; however, the current work suggests that segmenting the PVP into four major white matter tracts is useful for investigating the relationship between brain and behavior because we found that major tracts within the PVP had different relationships with learning.

Although we were able to demonstrate that some tracts were more related to learning than other tracts (tract-selectivity) and more related to one learning outcome relative to another learning outcome (task-selectivity), we found that the amount of variance explained by the microstructure of major white matter tracts was low. Finding a low amount of variance explained is remarkable because our approach employed a relaxed lasso regression that selected predictors that optimized the amount of variance explained. Our approach was to select the group of tracts whose white matter microstructure best explained drawing or visual recognition learning, yet even the best model could only explain approximately 12% of the variance (Table 1). These results suggest that the white matter microstructure of major white matter tracts can only explain a relatively small portion of individual variability in human learning. This is consistent with prior work demonstrating that the best prediction of future reading outcomes occurred when microstructural and functional activation measures were both included^74^. Future work focused on optimizing brain-behavior predictions will likely benefit from including brain function and other brain measurements in addition to microstructural measurements of major white matter tracts.

## Methods

### Participants

Adult participants (18-30 yrs., n = 60) were recruited through flyers posted on the Indiana University campus, online e-flyers, and through word-of-mouth. All participants were screened for neurological trauma, developmental disorders, and MRI contraindications. All participants were right-handed with English as their native language. Participants were compensated with a gift card for each session that they commenced. Data from participants were removed based on signal-to-noise (SNR), motion concerns, or other artifacts (see Magnetic resonance imaging data analyses) and, additionally, data from participants whose performance during training and/or testing revealed a lack of engagement were removed (see Learning rate calculations), leaving 48 subjects (age: *M* = 21.21 years, *SD* = 2.49 years, *Range* = [18.25, 29.75], 26 F, 22 M). All participants provided written informed consent and all procedures were approved by the Indiana University Institutional Review Board.

### Magnetic resonance image acquisition and procedure

Neuroimaging was performed at the Indiana University Imaging Research Facility, housed within the Department of Psychological and Brain Sciences with a 3-Tesla Siemens Prisma whole-body MRI using a 64-channel head coil. Participants were instructed to stay as still as possible during scanning and were allowed to watch a movie or listen to music of their choice during scanning.

T1-weighted anatomical volumes (i.e., t1w) were acquired using a Wave-CAIPI MP-RAGE pulse sequence (TR/TI/TE = 2300/900/3.47 ms, flip angle = 8°, acceleration factor = 3 in phase encoding direction × 3 in slice-selective direction, scan time = 1'14”), resolution = 1 mm isotropic. The T2-weighted anatomical volumes (i.e., t2w) were acquired with a 3D Wave-CAIPI pulse sequence (TR/TI/TE = 2300/900/3.47 ms, flip angle = 8°, acceleration factor = 3 in the phase encoding direction × 3 in slice-selective direction, scan time = 1'15”), resolution = 1 mm isotropic.

Diffusion data were collected using single-shot spin echo simultaneous multi-slice (SMS) EPI (transverse orientation, TE = 87.00 ms, TR = 3470 ms, flip angle = 78 degrees, isotropic 1.5 mm resolution; FOV = LR 210 mm × 192 mm × 138 mm; acquisition matrix MxP = 140 × 128. SMS acceleration factor = 4, interleaved). Diffusion data were collected at two diffusion gradient strengths, with 38 diffusion directions at b = 1000 s/mm^2^ and 37 directions at b = 2,500 s/mm^2^, as well as 5 images at b = 0 s/mm^2^, once in the AP fold-over direction (i.e., dwi-AP) and once in the PA fold-over direction (i.e., dwi-PA).

Within-session repeat scans were collected for each data type to ensure test-retest repeatability. For each participant, we collected two T1-weighted anatomical images, two T2-weighted anatomical images, two diffusion weighted images with AP phase-encoding, and two diffusion weighted images with PA phase-encoding.

### Magnetic resonance imaging data analyses

All analysis steps were performed using open and reproducible cloud services on the brainlife.io platform^75,76^, including ezBIDS^77^, except for the statistical analyses (see below) that were performed in Matlab R2019b using customized code. All data and analysis services are freely available on brainlife.io (Table 2). The code for the relaxed lasso regression is available here: https://github.com/svincibo/learning-white-matter. The code for all other statistical analyses is available here: https://github.com/svincibo/wml-wmpredictslearning.

**Table 2:** Data, description of analyses, and web-links to the open-source code and open cloud services used in the creation of this dataset can be viewed in their entirety here: 10.25663/brainlife.pub.36

Application	Github repository	Open Service DOI	Git Branch
Align T1 to ACPC Plane (HCP-based)	https://github.com/brain-life/app-hcp-acpc-alignment	10.25663/bl.app.99	1.4
Align T2 to ACPC Plane (HCP-based)	https://github.com/brain-life/app-hcp-acpc-alignment	10.25663/brainlife.app.116	1.4
Freesurfer Segmentation	https://github.com/brainlife/app-freesurfer	10.25663/brainlife.app.462	7.1.1
dMRI Preprocessing	https://github.com/brain-life/app-mrtrix3-preproc	10.25663/bl.app.68	1.7
NODDI model fitting	https://github.com/brainlife/app-noddi-amico	10.25663/brainlife.app.365	1.3
Tractography and Tensor model fitting	https://github.com/brain-life/app-mrtrix3-act	10.25663/brainlife.app.319	1.4
Tract Segmentation	https://github.com/brainlife/app-wmaSeg	10.25663/brainlife.app.188	3.9
Tract Cleaning	https://github.com/brainlife/app-removeTractOutliers	10.25663/brainlife.app.195	1.3
Tract Analysis Profiles	https://github.com/brain-life/app-tractanalysisprofiles	10.25663/brainlife.app.361	1.13
Tract Statistics	https://github.com/brainlife/app-tractographyQualityCheck	10.25663/brainlife.app.189	1.3

Anatomical images were aligned to the ACPC plane with an affine transformation using HCP preprocessing pipeline^78^ as implemented in the Align T1 to ACPC Plane (HCP-based) app on brainlife.io^79^ for t1w images and as implemented in the Align T2 to ACPC Plane (HCP-based) app on brainlife.io^79^ for t2w images. ACPC aligned images were then segmented using the Freesurfer 6.0^80^ as implemented in the Freesurfer App on brainlife.io^81^ to generate the cortical volume maps with labeled cortical regions according to the Destrieux 2009 atlas^82^.

All diffusion preprocessing steps were performed using the recommended MRtrix3 preprocessing steps^83^ as implemented in the MRtrix3 Preprocess App on brainlife.io^84^. AP phase-encoded and PA phase-encoded images were combined first and susceptibility- and eddy current-induced distortions as well as inter-volume subject motion were also corrected in this step. PCA denoising and Gibbs deringing procedures were then performed and the volumes were subsequently corrected for bias field and rician noise. Finally, the preprocessed dMRI data and gradients were aligned to each participant’s ACPC-aligned anatomical image using boundary-based registration (BBR) in FSL^85^.

Diffusion data were removed from the sample if the Signal-to-Noise Ratio (SNR) was less than 15 or if the Framewise Displacement (FD), a widely used measurement of head movement^86,87^, was greater than 2 mm or if an artifact was apparent. This resulted in a removal of 6 participants.

The microstructural properties of white matter tissue were estimated in a voxel-wise fashion based on preprocessed multi-shell dMRI data. We fit the diffusion tensor model (DTI) to the diffusion data to estimate the fractional anisotropy (FA), a summary measure of tissue microstructure that is thought to be related to the integrity of the myelin sheath and other tissue properties of major white matter tracts, such as axonal packing^88–90^.

Probabilistic tractography (PT) was used to generate streamlines. We used constrained spherical deconvolution (CSD) to model the diffusion tensor for tracking^91,92^. Tracking with the CSD model fit was performed probabilistically, using the tractography procedures provided by MRtrix3 Anatomically-constrained Tractography (ACT^93–95^; implemented in brainlife.io^96^. We generated 2 million streamlines at *L*max = 8 and a maximum curvature = 35 degrees, parameters that were optimized for our tractography needs. Streamlines that were shorter than 10 mm or longer than 200 mm were excluded. The tractogram was then segmented using the segmentation approach developed in^42^ and implemented on brailife.io^97^. All the files containing the processed data used in this manuscript are available here: 10.25663/brainlife.pub.36.

Streamlines that were more than 4 standard deviations away from the centroid of each tract and/or 4 standard deviations away from the relevant tract’s average streamline length were considered aberrant streamlines and were removed using the Remove Tract Outliers App on brainlife.io^98,99^.

Tract-profiles were generated for each major tract^99^ as well as the additional PVP tracts^42^ using the Tract Analysis Profiles app on brainlife.io^100^. We first resampled each streamline in a particular tract into 200 equally spaced nodes. At each node, we estimated the location of the tract’s ‘core’ by averaging the x, y, and z coordinates of each streamline at that node. We then estimated FA at each node of the core by averaging across streamlines within that node weighted by the distance of the streamline from the ‘core’. An average white matter measurement was obtained for each tract of interest by averaging across the central 160 nodes, excluding the first and last 20 nodes to avoid partial voluming effects.

### Behavioral procedures

Participants were asked to return for a behavioral session within one week of the neuroimaging session (Fig. 2). During the behavioral session, participants first performed a 30-min training session (i.e., Drawing training) followed by a visual recognition test (i.e., Visual recognition testing). An experimenter remained in the room with the participant throughout the behavioral session that was completed within 1 h. Code for behavioral procedures can be found here: https://github.com/svincibo/wml-beh.

Stimuli included 200 novel symbols. Using novel, unfamiliar symbols is a well-documented approach that controls for individual differences in pre-training symbol knowledge^101–104^ and allows for a cleaner manipulation of visual, auditory, and motor experience with those symbols. The design and selection criteria for these symbols is described in detail elsewhere^26^. The training required 40 symbols and the visual recognition test required an additional 40 distractor symbols, for a total of 80 symbols. The other 160 symbols were used for counterbalancing; the set of 80 symbols selected for each participant was counterbalanced across participants. Adobe Illustrator was used to create typed versions of these novel symbols. All symbols were in ‘typed’ form in black ink on a white background.

Drawing training: When participants arrived, they were seated at a desk with a digital Wacom writing tablet. Participants were asked to copy the novel symbols that we created using the tablet and instructed to make their productions as quickly and as accurately as possible. A Matlab script displayed one of the typed symbols at the top and center of the tablet screen and a box simultaneously appeared below the symbol into which participants were instructed to make their production of the symbol above. Only one symbol was displayed per trial and each trial lasted 4 s. Each block included 40 symbols and there were 10 back-to-back blocks, each containing the same 40 symbols. After completing 5 blocks, participants were given a mandatory 3-minute break to rest their hands and eyes before completing the final 5 blocks. The ordering of symbols within each block was randomized. Production duration time was measured for each symbol production trial as the number of seconds between the initial pen-down to the final pen-up.

Visual recognition testing: Participants were asked to perform an old/new recognition test immediately following the training session using an iMac computer and standard keyboard with a key labeled ‘yes’ and a different key labeled ‘no’. Participants first performed a practice session that consisted of individual letters of the alphabet and common shapes (e.g., square, triangle) and the participants were asked to press ‘yes’ for letters and ‘no’ for non-letters. The practice test helped orient participants to the testing context and lasted approximately 2 min. After the practice test, participants began the recognition test. During recognition testing, participants were presented with static, typed versions of the 40 learned symbols (i.e., target symbols) along with 40 symbols that were not presented to them during training (i.e., distractor symbols), one at a time and in random order. For each symbol, they were instructed to press ‘yes’ for symbols that they had practiced during training and ‘no’ for non-practiced symbols. Each trial consisted of only one symbol. Each trial began with a 500 ms fixation cross, followed by a 500 ms blank screen, and then a 25 ms stimulus presentation during which a stationary symbol was displayed in the center of the screen. After the stimulus presentation ended, the symbol was replaced by a noise mask until the participant responded or until the trial timed-out. Each trial timed-out after 1 second when participants received feedback that prompted them to respond faster in the next trial (i.e., “Too Slow!”). If the participant responded before the symbol was replaced by the noise mask, the program advanced to the blank screen until the trial time-out criteria was met before moving on to the next trial. Trials that reached the time-out limit were re-presented at the end of the test. Only trials with a participant response (i.e., trials that did not reach the 1-second time-out limit) were used for analyses. Reaction time and accuracy were measured.

### Learning calculations

Drawing learning: Learning rate of the sensorimotor task was calculated by first measuring the amount of time it took a participant to draw an unfamiliar symbol, i.e., the draw duration, and plotting this measurement across trials (Fig. 2b). Trials with a draw duration of 3 standard deviations above or below the within-participant mean were identified as outlier trials and removed. We tested both linear and double exponential models to model the change in draw duration over trials, given that both models have been used in the literature to model learning^8^. The double exponential models returned fits that were effectively linear despite aggressive efforts at bounding the fits, and the linear fits were good fits across participants. The learning rate was calculated across the 40 target symbols and 10 trials as the linear slope of draw duration over trials. The final learning rate for each participant was calculated by taking the slope across trials for that participant (Supplemental Fig. 3).

Visual recognition learning: Learning to visually recognize each symbol was calculated as the accuracy during visual recognition testing. Learning to visually recognize can be measured by their post-training recognition performance because participants were being tested on symbols that they had not been exposed to before they began drawing training. Participants with a visual recognition accuracy of 50% or lower, indicating that they were not performing above chance, were removed, resulting in the removal of 2 participants. We elected to use accuracy and not reaction time to measure visual recognition learning for three reasons: (1) an absence of a speed-accuracy trade-off (beta = 0.05, p = 0.57; Supplemental Fig. 4), (1) an absence of a ceiling effect for accuracy (0.55 < accuracy < 0.91), and (3) slightly greater individual variability captured by accuracy (SD = 0.09) than by reaction time (SD = 0.06).

### Statistical analyses

We were interested in understanding if white matter tracts within the posterior vertical pathway (pArc, TPC, MDLFang, MDLFspl) were more predictive of learning than tracts within the dorsal (SLF3, SLF1and2) and ventral (ILF, IFOF) cortices and, additionally, we were interested in understanding if the tracts that were strong predictors of sensorimotor learning were also predictive of visual perceptual learning. We included three additional control tracts, the vertical-occipital fasciculus (VOF), the frontal aslant tract (FAT), and the arcuate fasciculus (Arc) to control for the fact that the four PVP tracts are vertical tracts while the dorsal and ventral tracts are horizontal. The VOF, FAT, and Arc are vertical tracts that connect ventral cortex with dorsal cortex, but they do not directly connect ventral and parietal cortices. This resulted in a total of 22 tracts of interest, 11 tracts in the left hemisphere and 11 tracts in the right hemisphere.

We used relaxed lasso regression to directly compare among tracts (Fig. 2c). We entered the average FA for each tract as a predictor in a relaxed lasso model^60,61^, resulting in 22 potential predictors, one for each tract of interest (see **Methods: Magnetic resonance imaging data analyses** for the calculation of average FA of each tract). The relaxed lasso is a two-step procedure that first applies lasso followed by ordinary least squares. The ordinary lasso selects tracts that, together, explain the most variance in the response variable but is subject to a constraint on the size of the resulting coefficients, effectively shrinking the coefficient estimates. The relaxed lasso removes this shrinkage, de-biasing the coefficient estimates. All variables were standardized by dividing by their own variance to ensure that the magnitude of the beta estimates from the sensorimotor model and the visual recognition model were directly comparable. The best lasso model was selected based on leave-one-out cross-validation. We calculate the percent variance explained from the final relaxed lasso model, which uses only those predictors selected by the initial lasso screening in an ordinary least squares regression. This procedure was applied to the dependent variables of drawing learning and visual recognition learning separately, resulting in two final models, one for drawing learning and a second for visual recognition learning. We note that the relaxed lasso procedure tends to result in lower *R*^2^ values because it uses only a subset of all available predictors.

Additionally, we performed a series of simple linear (marginal) regression analysis to complement the results of the relaxed lasso analysis. Each model included one tract as the predictor and one behavioral measure as the response variable, resulting in 40 simple linear regression models for 20 tracts and 2 behavioral measures (Supplementary Information). We tested the significance of the beta-value assigned to the predictor in each model using *t*-test with alpha set to 0.05.

All analyses were applied to the additional repeat diffusion data to support replicability and reproducibility (see Magnetic resonance image acquisition and procedure). All statistical analyses were conducted using Matlab v9.11.10 (R2021b), except for the relaxed lasso analysis that was conducted using R v4.2.1 through RStudio 2022.02.1 build 461.

### Reporting summary

Further information on research design is available in the Nature Portfolio Reporting Summary linked to this article.

### Supplementary information

Supplementary Information

Reporting summary

## Data Availability

MRI data are available on brainlife.io at 10.25663/brainlife.pub.36.

## Code availability

The code for MRI data processing is available at 10.25663/brainlife.pub.36 (see Table 2 for more detail). The code for the relaxed lasso regression was conducted using R v4.2.1 through RStudio 2022.02.1 build 461 and is available here: https://github.com/svincibo/learning-white-matter. The code for all other statistical analyses were conducted using Matlab v9.11.10 (R2021b) and is available here: https://github.com/svincibo/wml-wmpredictslearning.
