# Supplementary material for: Associative white matter tracts selectively predict sensorimotor learning
Source: Commun Biol. 2024 Jun 22;7:762. doi: 10.1038/s42003-024-06420-1 (PMC11193801; doi:10.1038/s42003-024-06420-1)
Supplement: Supplementary file 1 — Supplementary Information [file 42003_2024_6420_MOESM1_ESM.pdf]

Supplementary Information

Supplemental materials: Results of relaxed lasso analysis using additional AFQ tracts

To ensure that our results were not affected by our hypothesis driven selection of 22 white matter tracts, we conducted our analysis with an extended set of white matter tracts. We included the set of tracts segmented by AFQ [1] with the addition of the four posterior vertical tracts [2] from both hemispheres for a total of 34 tracts. This analysis, therefore, Included the same 22 tracts as in the main text, with the addition of 12 added tracts: uncinate, thalamic radiation, callosum forceps major, callosum forceps minor, cingulum, and corticospinal tract. For drawing learning, results were largely consistent with the findings using the hypothesis-driven entry of 22 tracts into the relaxed lasso regression: the left pArc and left SLF 3 were identified as predictors of drawing learning (**Supplemental Table 1**). However, for visual recognition learning, the relaxed lasso regression failed to identify any tracts that predicted visual recognition learning where the analysis with the hypothesis-driven entry of 22 tracts into the relaxed lasso revealed the left MDLFspl in the original and repeat dataset.

Supplemental Table 1. *Relaxed lasso regression results using the set of tracts segmented by AFQ in addition to the four posterior vertical tracts included in the main text.*

**Response Variable**	**Predictor**	***β***	***S.E.***	***R^2***
Drawing learning	Left pArc	0.2803	0.3219	0.0924
Drawing learning (repeat dataset)	Left pArc	0.2731	0.2926	0.2210
	Left SLF3	0.2170	0.2960	-
	Right fronto-thalamic radiation	-0.4252	0.3247	-
Visual recognition learning	-	-	-	-
Visual recognition learning (repeat dataset)	-	-	-	-

Supplemental materials: Simple linear regression to identify tracts that independently predict drawing and recognition learning

Simple linear (marginal) regression analyses evaluated the relationship between each learning outcome and the microstructure of each tract separately to identify individual tracts (not groups of tracts) that were able to explain a significant amount of variance in learning outcomes. A simple linear regression analysis was conducted for each white matter tract that was included in the relaxed lasso regressions described in the main text and for each learning outcome, resulting in 22 simple linear regressions with drawing learning as the dependent variable and another 22 with visual recognition learning as the dependent variable. Model significance was evaluated using an F-test with $p < 0.05$. Results from all significant regressions are reported below and in the **Supplemental Table 2** and visually displayed with 95% bootstrapped confidence intervals in the **Supplemental Figure 1** with individual data for all participants displayed in **Supplemental Figure 2**. Results from all regressions, including non-significant results, are displayed in the **Supplemental Table 3**.

Learning to draw novel symbols: left pArc and left SLF3

The results of the simple linear regression analyses identified the microstructure of only 2 tracts that significantly predicted drawing learning: the left pArc and the left SLF3 (**Supplemental Table 2**). Consistent with the results of the relaxed lasso regression, the relationship between each tract and drawing learning was positive, such that participants with higher FA were participants who were the quickest at learning to draw the novel symbols (**Supplemental Figure 3**), however neither result passed a Bonferonni correction for multiple comparisons, $p < 0.05/22$, i.e., $p < 0.0023$. There were no other tracts that significantly predicted drawing learning, all *p*s > 0.05.

Visual recognition learning: no significant tracts

The simple linear regression analyses did not identify any tract that individually predicted visual recognition learning in either the original or repeat data set, all *p*s > 0.05.

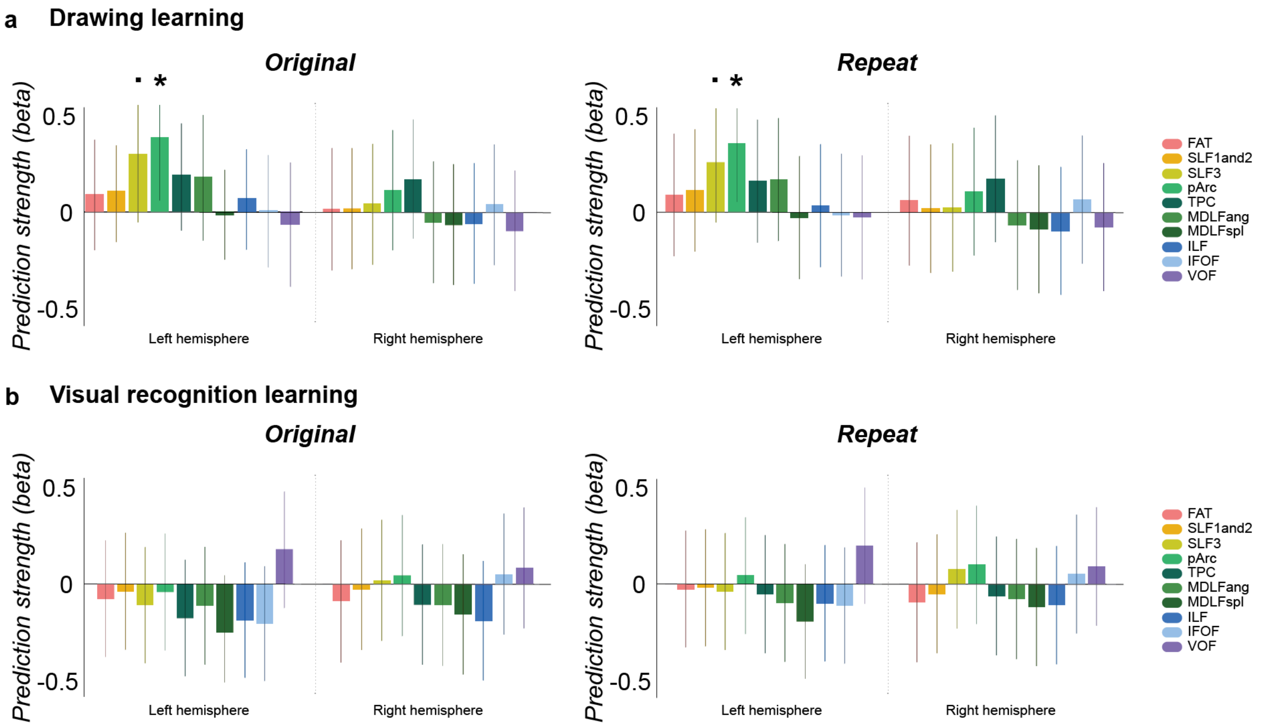

Supplemental Figure 1. Simple linear (marginal) regression results: Prediction strength of tract microstructure for drawing and visual recognition learning. **a.** Drawing learning. The left pArc and left SLF3 significantly predicted drawing learning. **b.** Visual recognition learning. There were no tracts that significantly predicted visual recognition learning. Frontal aslant (FAT); superior longitudinal fasciculus, 1st and 2nd segment (SLF1and2); superior longitudinal fasciculus, 3rd segment (SLF3); posterior arcuate fasciculus (pArc); temporal-parietal connection (TPC); middle longitudinal fasciculus connection to the angular gyrus (MDLFang); middle longitudinal fasciculus connection to the superior parietal lobe (MDLFspl); inferior longitudinal fasciculus (ILF); inferior fronto-occipital fasciculus (IFOF); vertical occipital fasciculus (VOF). Error bars represent 95% bootstrapped confidence intervals with 10,000 iterations. See **Supplemental Figure 2** for simple linear regression results for individual data on all participants ˙, $p < 0.10$; *, $p < 0.05$.

Supplemental Table 2. *Relationships between one learning outcome and one tract using simple linear regression.*

**Response Variable**	**Predictor**	***β***	***S.E.***	***p***	
Drawing learning	Left pArc	0.3505	0.1381	0.0146	*
	Left SLF3	0.2745	0.1418	0.0590	.
Drawing learning (repeat dataset)	Left pArc	0.3308	0.1391	0.0216	*
	Left SLF3	0.2396	0.1432	0.1010	.
Visual recognition learning	-	-	-	-	
Visual recognition learning (repeat dataset)	-	-	-	-	

Note: *All tracts were tested for each learning outcome; however, only significant simple linear regression models are shown here for simplicity. *, p < 0.05; . , p < 0.10.*

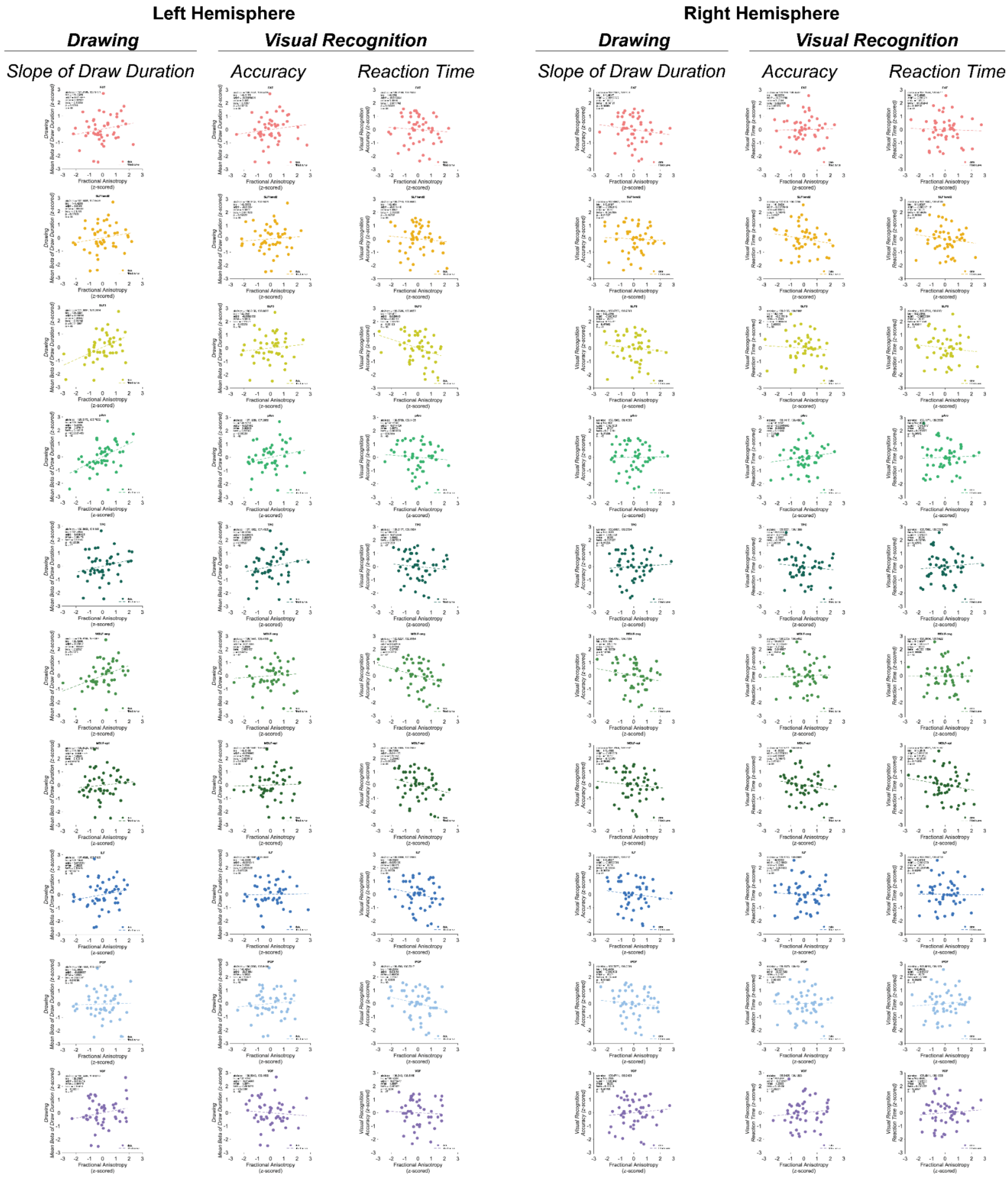

Supplemental Figure 2. Simple linear (marginal) regression results displayed for each tract separately.

Supplemental Table 3.
Relationships between one learning outcome and one tract using simple linear regression.

Response Variable	Predictor	β	S.E.	p	
Drawing learning					
	Left pArc	0.3505	0.1381	0.0146	*
	Left SLF3	0.2745	0.1418	0.0590	.
	Left FAT	0.0827	0.1469	0.5765	
	Left SLF1and2	0.0937	0.1468	0.5261	
	Left TPC	0.1701	0.1469	0.2530	
	Left MDLFang	0.1617	0.1471	0.2777	
	Left MDLFspl	-0.0201	0.1474	0.8922	
	Left Arc	0.2057	0.1443	0.1607	
	Left ILF	0.0593	0.1472	0.6885	
	Left IFOF	0.0042	0.1474	0.9776	
	Left VOF	-0.0567	0.1489	0.7052	
	Right pArc	0.1063	0.1467	0.4720	
	Right SLF3	0.0383	0.1473	0.7957	
	Right FAT	0.0134	0.1491	0.9286	
	Right SLF1and2	0.0164	0.1474	0.9119	
	Right TPC	0.1613	0.1455	0.2734	
	Right MDLFang	-0.0506	0.1489	0.7356	
	Right MDLFspl	-0.0624	0.1472	0.6736	
	Right Arc	0.1514	0.1457	0.3043	
	Right ILF	-0.0571	0.1472	0.6997	
	Right IFOF	0.0358	0.1474	0.8091	
	Right VOF	-0.0923	0.1468	0.5328	
Drawing learning (repeat dataset)					
	Left pArc	0.3308	0.1391	0.0216	*
	Left SLF3	0.2396	0.1432	0.1010	.
	Left FAT	0.0841	0.1469	0.5699	
	Left SLF1and2	0.1041	0.1466	0.4816	
	Left TPC	0.1506	0.1474	0.3124	
	Left MDLFang	0.1574	0.1472	0.2908	
	Left MDLFspl	-0.0273	0.1474	0.8541	
	Left Arc	0.1785	0.1451	0.2248	
	Left ILF	0.0310	0.1474	0.8344	
	Left IFOF	-0.0140	0.1474	0.9247	
	Left VOF	-0.0247	0.1490	0.8692	
	Right pArc	0.0959	0.1468	0.5166	
	Right SLF3	0.0201	0.1474	0.8919	
	Right FAT	0.0531	0.1489	0.7228	
	Right SLF1and2	0.0149	0.1474	0.9199	
	Right TPC	0.1551	0.1457	0.2927	
	Right MDLFang	-0.0603	0.1488	0.6871	
	Right MDLFspl	-0.0784	0.1470	0.5963	
	Right Arc	0.1447	0.1459	0.3264	
	Right ILF	-0.0854	0.1469	0.5637	
	Right IFOF	0.0579	0.1472	0.6961	
	Right VOF	-0.0688	0.1471	0.6422	
Visual recognition learning					
	Left pArc	-0.0409	0.1473	0.7826	
	Left SLF3	-0.1076	0.1466	0.4666	
	Left FAT	-0.0740	0.1470	0.6171	
	Left SLF1and2	-0.0376	0.1473	0.8000	

	Left TPC	-0.1727	0.1468	0.2457
	Left MDLFang	-0.1101	0.1482	0.4613
	Left MDLFspl	-0.2448	0.1430	0.0936
	Left Arc	-0.0981	0.1467	0.5069
	Left ILF	-0.1832	0.1450	0.2126
	Left IFOF	-0.2017	0.1444	0.1692
	Left VOF	0.1738	0.1468	0.2426
	Right pArc	0.0401	0.1473	0.7869
	Right SLF3	-0.0164	0.1474	0.9121
	Right FAT	-0.0853	0.1485	0.5687
	Right SLF1and2	-0.0269	0.1474	0.8561
	Right TPC	-0.1020	0.1467	0.4903
	Right MDLFang	-0.1037	0.1483	0.4880
	Right MDLFspl	-0.1494	0.1458	0.3107
	Right Arc	-0.1029	0.1467	0.4863
	Right ILF	-0.1802	0.1450	0.2204
	Right IFOF	0.0469	0.1473	0.7516
	Right VOF	0.0773	0.1470	0.6015
Visual recognition learning (repeat dataset)				
	Left pArc	0.0424	0.1473	0.7747
	Left SLF3	-0.0387	0.1473	0.7940
	Left FAT	-0.0272	0.1474	0.8546
	Left SLF1and2	-0.0196	0.1474	0.8947
	Left TPC	-0.0527	0.1489	0.7249
	Left MDLFang	-0.0970	0.1484	0.5166
	Left MDLFspl	-0.1909	0.1447	0.1936
	Left Arc	-0.0393	0.1473	0.7909
	Left ILF	-0.0992	0.1467	0.5023
	Left IFOF	-0.1091	0.1466	0.4606
	Left VOF	0.1938	0.1463	0.1919
	Right pArc	0.0944	0.1468	0.5233
	Right SLF3	0.0724	0.1471	0.6249
	Right FAT	-0.0906	0.1485	0.5449
	Right SLF1and2	-0.0501	0.1473	0.7352
	Right TPC	-0.0595	0.6879	0.1472
	Right MDLFang	-0.0764	0.1486	0.6098
	Right MDLFspl	-0.1141	0.1465	0.4399
	RightArc	-0.0787	0.1470	0.5948
	Right ILF	-0.1058	0.1466	0.4741
	Right IFOF	0.0484	0.1473	0.1473
	Right VOF	0.0873	0.1469	0.5553

*NOTE: *, p < 0.05; . , p < 0.10.*

Supplemental materials: Participants learned to draw and visually recognize symbols during training

To ensure that participants did, in fact, learn to draw and also to visually recognize symbols during the training session, we performed one-sample *t*-test on the sensorimotor learning variable (i.e., slope of draw duration across trials) to confirm that the slope was less than zero (i.e., negative) and also on the visual recognition learning variable (i.e., accuracy) to confirm that it was above chance (i.e., 50%). These analyses confirmed that participants did experience an increase in their ability to draw the symbols throughout the training session and that they also learned to visually recognize the symbols throughout the training session.

Learning to draw novel symbols

Participants became faster at drawing symbols throughout the drawing training session, suggesting that participants learned to draw novel symbols during training (**Supplemental Figure 3a**). We measured the drawing duration for each symbol drawing trial throughout the 30-minute training session and calculated the slope of draw duration over trials. A one-sample *t*-test on the slope of each participant's drawing durations confirmed that participants' learning slopes (*M* = -8.6e-4, *SD* = 1.2e-3) were significantly less than zero, *t*(47) = 10.05, *p* = 5.06e-4, demonstrating that participants spent less time drawing symbols with each trial that they completed. Additionally, a density histogram of participants' learning slopes demonstrated that the learning slopes for most participants was negative (**Supplemental Figure 3a**).

The speed with which participants drew the symbols increased during drawing training, suggesting that participants were learning how to draw the novel symbols during the training session. To our knowledge, learning to draw novel symbols during drawing training has not yet been demonstrated in adult subjects, although it is certainly an intuitive result. One prior work in adults has demonstrated that the drawn productions of common objects became more recognizable with increased practice drawing those objects [3], consistent with the notion that drawing practice improves drawing ability. Additionally, training studies using other sensorimotor learning tasks report similar results: practice with piano playing improves piano playing [4,5] and practice juggling improves juggling [6]. Notably, increases in the speed of drawing occurred without explicit pressure to learn to draw the symbols. We encouraged participants to draw symbols as quickly and as accurately as possible and they were aware that they would be tested on their ability to recognize the symbols after drawing, but they were not aware that we were measuring the duration of their drawings to estimate drawing learning.

Visual recognition learning

Participants performed the visual recognition test with above-chance accuracy, suggesting that participants learned to visually recognize the symbols during production training (**Supplemental Figure 3b**). A one-sample *t*-test confirmed that participants' accuracy during the recognition test (*M* = 0.76, *SD* = 0.09) was significantly above chance (i.e., 50%), *t*(47) = 12.12, *p* = 4.49e-16, demonstrating that their responses during recognition testing were not likely due to random guessing. Additionally, we visualized reaction times for each participant (**Supplemental Figure 3b**) and performed a simple linear regression that revealed no significant relationship between accuracy and reaction time, suggesting the absence of a speed-accuracy trade-off (R^2 = 0.048, *beta* = 0.05, *p* = 0.57) (**Supplemental Figure 4a**).

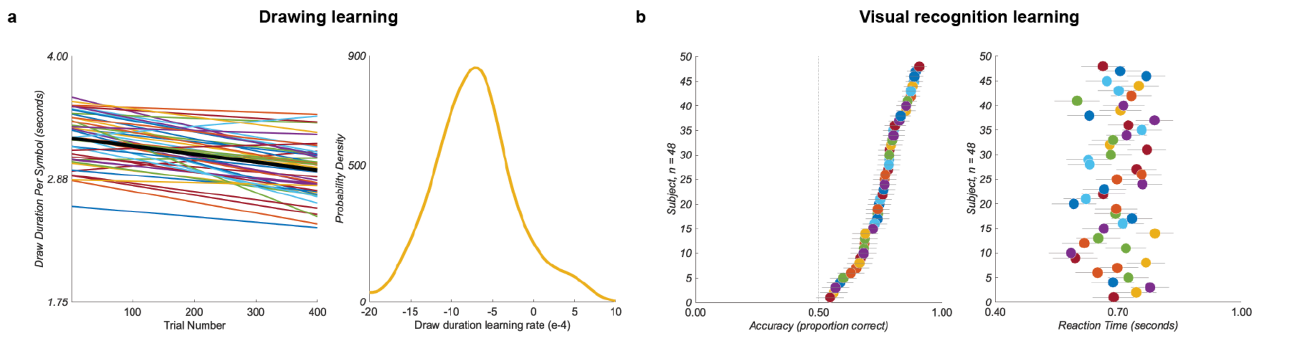

Supplemental Figure 3. Behavioral results. **a. Participants learned to draw the symbols.** The draw duration across symbols decreased over the course of the production training (left), $t(47) = 10.05$, $p = 5.06e\text{-}4$. The black line is the slope of draw duration across trials for all participants. Each non-black line is the linear fit of draw duration across trials for a single participant. The slope of the linear fit of draw duration across trials was negative for nearly all participants with a probability density centered around -8e-4, indicating that the majority of participants learned to draw the novel symbols during training. **b. Participants learned to recognize the symbols.** Participants visually recognized the symbols with above-chance accuracy after production training, $t(47) = 12.12$, $p = 4.49e\text{-}16$, suggesting that participants learned to recognize the trained symbols. Each dot represents the proportion correct during the visual recognition test for each individual participant. Average reaction time is also displayed for each individual participant. Error bars represent standard error across correct trials. For both **a** and **b**, individual subjects are color coded and represented only once per plot.

Prior work has demonstrated that drawing is beneficial for visual recognition learning [7–12], and our results are consistent with the results of these prior studies. In the current study, participants were asked to draw symbols that they had never seen before and were then tested on their ability to visually recognize those symbols. Participants recognized symbols with above chance accuracy after drawing training (**Supplemental Figure 3b**), suggesting that the drawing training contributed to visual recognition learning. However, we did not explicitly manipulate the drawing training and are, therefore, unable to conclude that the drawing training affected visual recognition learning in this study. The drawing training was a copy task in which a typed symbol remained on the screen as a model while participants copied the symbol. It is possible that the above chance recognition accuracy after drawing training resulted from exposure to the model symbol and not the drawing training. Because these were novel symbols, any exposure to the symbols would be expected to lead to above-chance recognition accuracy.

Although it is possible that the above chance recognition accuracy observed after training resulted from only the visual experience of seeing the model symbol, it is highly likely that some of the recognition learning observed after training resulted from drawing. First, prior work has demonstrated that drawing experience facilitates visual recognition of the items that were drawn more than visually perceiving typed symbols [9–14] and more than other motor activities, such as typing [9,10]. Second, although participants may have learned to visually recognize the symbols from seeing the model symbol, prior research has demonstrated that the act of drawing the symbol has its own effect on visual recognition. In pre-post training studies that have included a model symbol for copying, drawing the symbol beneath a model symbol increased visual recognition more than watching someone else draw the symbol beneath a model symbol, drawing the symbol using a pen without ink beneath a model symbol, watching a symbol unfold on a screen as if being drawn beneath a model symbol, or viewing a static handwritten version of the symbol beneath a model symbol [12,14]. Thus, although we did not include a control condition in this study to determine that the recognition learning was not simply a consequence

of exposure to the model symbol during training, prior work using similar study designs suggests that at least some of the recognition learning resulted from drawing.

No significant relationship between learning to draw and learning to visually recognize

A simple linear regression was used to determine if the learning rate of symbol drawing was related to visual recognition learning. The predictor was the slope of the draw duration across trials during production training and the response variable was accuracy. We also tested to see if the slope of draw duration across trials was related to reaction time. We tested the significance of the model using an *F*-test with alpha set to 0.05.

We conducted a simple linear regression analysis to determine if the participants who were quicker at learning to draw symbols were also the participants who were better able to recognize the symbols after drawing. Surprisingly, we observed no significant relationship between learning to draw and learning to visually recognize symbols. Drawing learning was not related to visual recognition learning. A simple linear regression revealed that the learning slope of symbol drawing duration was not a significant predictor of visual recognition learning, as measured by either accuracy (R^2 = 0.019, *beta* = 0.05, *p* = 0.72) or reaction time (R^2 = 0.014, *beta* = -0.07, *p* = 0.55) (**Supplemental Figure 4b**). We followed these results with a linear mixed-effects analysis that included random effects for symbol and participant and found similar results. The addition of random effects for symbol and participant significantly improved the model fits; however, the relationship between draw duration slope on visual recognition performance remained non-significant, all *p*s > 0.05.

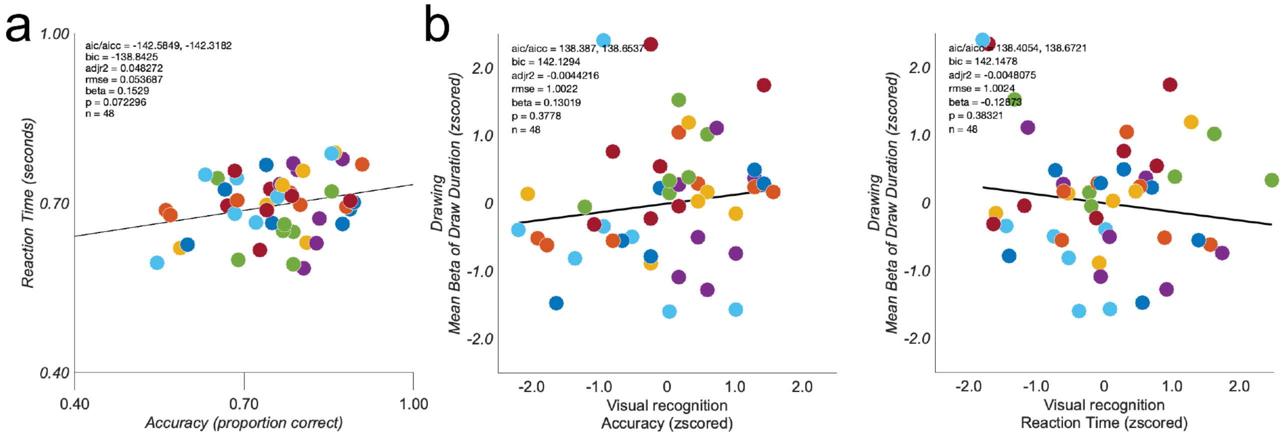

Supplemental Figure 4. Relationships among behavioral measurements. **a. Visual recognition learning: no speed-accuracy trade-off.** The relationship between accuracy and reaction time was not significantly different from zero, *beta* = 0.05, *p* = 0.57, suggesting that no speed-accuracy trade-off occurred. **b. Non-significant relationship between drawing and recognition learning.** The slope of draw duration over trials was not related to visual recognition accuracy, *beta* = 0.13, *p* = 0.38, or reaction time, eta*b* = -0.13, *p* = 0.38, suggesting that participants who were better at learning to draw the symbols were not the same participants who were better at recognition.

Supplemental materials: J-test

Results of the J-test were consistent with the results of the Cox test and revealed that adding the fitted values from the original recognition model as an additional predictor in the drawing learning model to create a new transfer model did not significantly improve the model fit beyond the fit of the original drawing learning model fit, $t = 0.227$, $p = 0.821$, and the fit of the original recognition learning model was not significantly better than the fit of the transfer model, $t = 1.686$, $p = 0.099$.

In the repeat dataset, again, transfer model did not significantly improve the model fit beyond the fit of the original drawing learning model fit, $t = 0.2169$, $p = 0.8293$, and the fit of the original recognition learning model was not significantly better than the fit of the transfer model, $t = 1.5153$, $p = 0.1368$.

Supplemental materials: Cox-test and J-test for reaction time measurement of visual recognition learning

The analysis reported in the main text uses the accuracy measurement for visual recognition learning. Here, we report the analyses using the reaction time measurement for visual recognition learning. More specifically, we demonstrate that the predictors selected by the relaxed lasso (RL) regression for drawing learning do not transfer to visual recognition learning when visual recognition learning was measured using reaction time. This result is consistent with the results reported in the main text for visual recognition learning measured as accuracy.

The first analysis was exactly the same as the first analysis reported in the main text, an RL regression with cross-validation to identify the set of predictors that best explains variance in drawing learning. The selected model included two predictors corresponding to the L pArc and L SLF3, with an OLS R^2 = 0.1180 (**Table 1**).

The second analysis was the same as the second analysis reported in the main text, except that we measured visual recognition learning using reaction time instead of accuracy. We performed an RL regression with cross-validation to identify the set of predictors that best predicted visual recognition learning (using reaction time). The selected model included no predictors, indicating that an intercept model was the best model for predicting visual recognition learning as measured using reaction time.

The third analysis used the Cox-test, as in the main text, to determine if the predictors originally selected for drawing learning (i.e., with predictors corresponding to the left pArc and left SLF3) might be able to explain additional variance in visual recognition learning beyond the variance explained by the predictors selected in the original recognition model (i.e., an intercept only model). Results demonstrated that the model selected for drawing learning (i.e., with predictors corresponding to the left MDLFspl and the, left TPC) was a better fit for visual recognition learning than the transfer model (i.e., an intercept only model), $z = -1.293$, $p = 2e\text{-}16$.

The J-test provided complementary results. Adding the fitted values from the original recognition model as an additional predictor in the drawing learning model to create a new transfer model could not

significantly improve the model fit beyond the fit of the original drawing learning model fit, by definition, because the transfer model was an intercept only model in this case. Adding the fitted values from the original drawing learning model as an additional predictor in the visual recognition model did not significantly improve the model fit beyond the original visual recognition model, $t = 1.595$, $p = 0.118$.

Results in the repeat dataset were consistent with these results. The first analysis selected the L pArc and L SLF3 as the best predictors of drawing learning, as originally reported in the main text, with an OLS $R^2 = 0.0969$ (**Table 1**). The second analysis selected the model that best predicted visual recognition learning based on the reaction time measurement, finding that the intercept was the best fitting model. Finally, the third analysis used the Cox-test to demonstrate that the model selected for drawing learning (i.e., with predictors corresponding to the left MDLFspl and the, left TPC) was a better fit for visual recognition learning than the transfer model (i.e., an intercept only model), $z = -1.51e13$, $p = 2e-16$. The J-test revealed similar results. Adding the fitted values from the original drawing learning model as an additional predictor in the visual recognition learning model did not significantly improve the model fit beyond the original visual recognition model, $t = 1.439$, $p = 0.157$.

References

1. Yeatman, J. D., Dougherty, R. F., Myall, N. J., Wandell, B. A. & Feldman, H. M. Tract profiles of white matter properties: automating fiber-tract quantification. *PLoS One* **7**, e49790 (2012).
2. Bullock, D. *et al.* Associative white matter connecting the dorsal and ventral posterior human cortex. *Brain Struct. Funct.* (2019) doi:10.1007/s00429-019-01907-8.
3. Fan, Yamins & Turk-Browne. Common object representations for visual recognition and production. *CogSci* (2015).
4. Bassett, D. S., Yang, M., Wymbs, N. F. & Grafton, S. T. Learning-induced autonomy of sensorimotor systems. *Nat. Neurosci.* **18**, 744–751 (2015).
5. Engel, A. *et al.* Learning piano melodies in visuo-motor or audio-motor training conditions and the neural correlates of their cross-modal transfer. *Neuroimage* **63**, 966–978 (2012).
6. Scholz, J., Klein, M. C., Behrens, T. E. J. & Johansen-Berg, H. Training induces changes in white-matter architecture. *Nat. Neurosci.* **12**, 1370–1371 (2009).
7. Fan, J. E., Yamins, D. L. K. & Turk-Browne, N. B. Common Object Representations for Visual Production and Recognition. *Cogn. Sci.* **42**, 2670–2698 (2018).
8. Wammes, J. D., Jonker, T. R. & Fernandes, M. A. Drawing improves memory: The importance of multimodal encoding context. *Cognition* **191**, 103955 (2019).
9. Longcamp, M., Zerbato-Poudou, M.-T. & Velay, J.-L. The influence of writing practice on letter recognition in preschool children: a comparison between handwriting and typing. *Acta Psychol.* **119**, 67–79 (2005).
10. Kiefer, M. *et al.* Handwriting or Typewriting? The Influence of Pen- or Keyboard-Based Writing Training on Reading and Writing Performance in Preschool Children. *Adv. Cogn. Psychol.* **11**, 136–146 (2015).
11. Li, J. X. & James, K. H. Handwriting generates variable visual output to facilitate symbol learning. *J. Exp. Psychol. Gen.* **145**, 298–313 (2016).
12. Vinci-Booher, S., James, T. W. & James, K. H. Visual-motor contingency during symbol production

contributes to short-term changes in the functional connectivity during symbol perception and long-term gains in symbol recognition. *Neuroimage* **227**, 117554 (2020).

13. Zemlock, D., Vinci-Booher, S. & James, K. H. Visual–motor symbol production facilitates letter recognition in young children. *Read. Writ.* **31**, 1255–1271 (2018).
14. Vinci-Booher, S., Sehgal, N. & James, K. H. Visual and Motor Experiences of Handwriting Independently Contribute to Gains in Visual Recognition. (2018).
