# Supplementary material for: Associative white matter tracts selectively predict sensorimotor learning
Source: Commun Biol. 2024 Jun 22;7:762. doi: 10.1038/s42003-024-06420-1 (PMC11193801; doi:10.1038/s42003-024-06420-1)
Supplement: Supplementary file 2 — Reporting summary [file 42003_2024_6420_MOESM2_ESM.pdf]

nature portfolio

Corresponding author(s): Franco Pestilli

Last updated by author(s): May 15, 2024

Reporting Summary

Nature Portfolio wishes to improve the reproducibility of the work that we publish. This form provides structure for consistency and transparency in reporting. For further information on Nature Portfolio policies, see our Editorial Policies and the Editorial Policy Checklist.

Statistics

For all statistical analyses, confirm that the following items are present in the figure legend, table legend, main text, or Methods section.

n/a	Confirmed	
☐	☒	The exact sample size (n) for each experimental group/condition, given as a discrete number and unit of measurement
☐	☒	A statement on whether measurements were taken from distinct samples or whether the same sample was measured repeatedly
☐	☒	The statistical test(s) used AND whether they are one- or two-sided<br>*Only common tests should be described solely by name; describe more complex techniques in the Methods section.*
☐	☒	A description of all covariates tested
☐	☒	A description of any assumptions or corrections, such as tests of normality and adjustment for multiple comparisons
☐	☒	A full description of the statistical parameters including central tendency (e.g. means) or other basic estimates (e.g. regression coefficient) AND variation (e.g. standard deviation) or associated estimates of uncertainty (e.g. confidence intervals)
☒	☐	For null hypothesis testing, the test statistic (e.g. F, t, r) with confidence intervals, effect sizes, degrees of freedom and P value noted<br>*Give P values as exact values whenever suitable.*
☒	☐	For Bayesian analysis, information on the choice of priors and Markov chain Monte Carlo settings
☒	☐	For hierarchical and complex designs, identification of the appropriate level for tests and full reporting of outcomes
☒	☐	Estimates of effect sizes (e.g. Cohen's d, Pearson's r), indicating how they were calculated

Our web collection on statistics for biologists contains articles on many of the points above.

Software and code

Policy information about availability of computer code

Data collection	Data collection made use of custom Matlab code that is provided on github. The exact online addresses are provided in the main text.
Data analysis	Data collection made use of custom Matlab code and R code that are provided on github. The exact online addresses are provided in the main text.

For manuscripts utilizing custom algorithms or software that are central to the research but not yet described in published literature, software must be made available to editors and reviewers. We strongly encourage code deposition in a community repository (e.g. GitHub). See the Nature Portfolio guidelines for submitting code & software for further information.

Data

Policy information about availability of data

All manuscripts must include a data availability statement. This statement should provide the following information, where applicable:

- Accession codes, unique identifiers, or web links for publicly available datasets
- A description of any restrictions on data availability
- For clinical datasets or third party data, please ensure that the statement adheres to our policy

All raw data and data derivatives are provided on brainlife.io. The exact online address is provided in the main text.

Research involving human participants, their data, or biological material

Policy information about studies with human participants or human data. See also policy information about sex, gender (identity/presentation), and sexual orientation and race, ethnicity and racism.

Reporting on sex and gender	Sex is reported in the participants section based on self-report. It was not included as a variable in any of the analyses.
Reporting on race, ethnicity, or other socially relevant groupings	No socially constructed categories were used as they were not considered relevant to the research questions.
Population characteristics	See below.
Recruitment	Participants were recruited through flyers posted on the Indiana University campus, online e-flyers, and through word-of-mouth.
Ethics oversight	Indiana University

Note that full information on the approval of the study protocol must also be provided in the manuscript.

Field-specific reporting

Please select the one below that is the best fit for your research. If you are not sure, read the appropriate sections before making your selection.

☐ Life sciences ☒ Behavioural & social sciences ☐ Ecological, evolutionary & environmental sciences

For a reference copy of the document with all sections, see nature.com/documents/nr-reporting-summary-flat.pdf

Behavioural & social sciences study design

All studies must disclose on these points even when the disclosure is negative.

Study description	individual differences; quantitative
Research sample	The research sample was selected from the city of Bloomington, Indiana. The recruited sample included 60 participants. The final sample after attrition and data removal (see below) included 48 participants, ranging in age from 18- 30 years old with 26 female and 22 male participants. This sample is representative of the general healthy/typical population in terms of a biological relationship with learning.
Sampling strategy	The sample was randomly selected. Sample sizes were chosen based on prior research with similar procedures and goals and were considered sufficient for the current work.
Data collection	Data were collected using an MRI machine and also using a computer, keyboard, and digital Wacom tablet to collect drawing movements. An experimenter was present in the room while participants used the computer, keyboard, and Wacom set-up. The experimenter was not blind to the hypothesis; however, it was impossible for them to access the brain data results and, therefore, the experimenters had no way of knowing each participant's brain results and could not bias their administration of the procedure with the computer, keyboard, and Wacom tablet.
Timing	Data collection started August 1, 2021 and ended June 1, 2022.
Data exclusions	A total of 12 participants were recruited but not included in the analyses, resulting in a final sample size of 48. Participants were lost to attrition, e.g., not finishing MRI procedure or not coming to the second day (n = 4). Participants were removed if the signal-to-noise (SNR) of their MRI data indicated that the data were of insufficient quality (n = 6) and if the results of their behavioral assessments indicated a lack of engagement, i.e., performance at or below chance (n = 2).
Non-participation	No participants declined th participate. A total of 4 participants dropped out of the study, either because they did not want to finish the MRI session or they were unable to attend the second day of behavioral assessments due to an unforeseen event.
Randomization	Participants were not allocated into experimental groups.

Reporting for specific materials, systems and methods

We require information from authors about some types of materials, experimental systems and methods used in many studies. Here, indicate whether each material, system or method listed is relevant to your study. If you are not sure if a list item applies to your research, read the appropriate section before selecting a response.

Materials & experimental systems

n/a	Involved in the study
☒	☐ Antibodies
☒	☐ Eukaryotic cell lines
☒	☐ Palaeontology and archaeology
☒	☐ Animals and other organisms
☒	☐ Clinical data
☒	☐ Dual use research of concern
☒	☐ Plants

Methods

n/a	Involved in the study
☒	☐ ChIP-seq
☒	☐ Flow cytometry
☐	☒ MRI-based neuroimaging

Magnetic resonance imaging

Experimental design

Design type: Anatomical and diffusion imaging only, i.e., no functional imaging

Design specifications: N/A

Behavioral performance measures: N/A

Acquisition

Imaging type(s): structural, diffusion

Field strength: 3T

Sequence & imaging parameters: Structural: Wave-CAIPI MP-RAGE pulse sequence, FOV: 256x256x256 mm3, slice thickness: 1.0 mm, orientation: transversal, TE: 3.47 s, TR: 2300.00 ms, flip angle: 8 degrees; Diffusion: single-shot spin echo simultaneous multi-slice (SMS) EPI, FOV: LR 210x192x138 mm3; acquisition matrix MxP: 140 x 128, slice thickness: 1.5 mm, orientation: transverse, TE: 87.00 ms, TR: 3470 ms, flip angle: 78 degrees.

Area of acquisition: Whole brain scan.

Diffusion MRI ☒ Used ☐ Not used

Parameters: 38 diffusion directions at b = 1,000 s/mm2 and 37 directions at b = 2,500 s/mm2, as well as 5 images at b = 0 s/mm2, once in the AP fold-over direction (i.e., dwi-AP) and once in the PA fold-over direction (i.e., dwi-PA)

Preprocessing

Preprocessing software: A variety of preprocessing software was used. Details about all preprocessing parameters are publicly available here: https://brainlife.io/pub/634f271cfa262bbde2b493f5.

Normalization: The anatomical images were aligned to the ACPC plane using the MNI152_T1_1mm template.

Normalization template: The template used for aligning the anatomicals to ACPC plane was MNI152_T1_1mm template.

Noise and artifact removal: *Describe your procedure(s) for artifact and structured noise removal, specifying motion parameters, tissue signals and physiological signals (heart rate, respiration).*

Volume censoring: *Define your software and/or method and criteria for volume censoring, and state the extent of such censoring.*

Statistical modeling & inference

Model type and settings: 2 relaxed lasso regression models. The first model used the slope of draw duration over trials as the response variable and selected from a group of 22 predictors that were the average FA of each of the 22 white matter tracts tested. The second model used the visual recognition accuracy score as the response variable and the selected from the same group of 22 predictors as the first model. For both models, there was one observation per participant.

Effect(s) tested: tested the relationship between average fractional anisotropy of major white matter tracts and (1) the change in drawing duration of unknown symbols and (2) the accuracy of visual recognition of the previously unknown symbols after drawing learning

Specify type of analysis: ☒ Whole brain ☐ ROI-based ☐ Both

Statistic type for inference
(See Eklund et al. 2016)

N/A

Correction

N/A

Models & analysis

n/a	Involved in the study
☒	☐ Functional and/or effective connectivity
☒	☐ Graph analysis
☒	☐ Multivariate modeling or predictive analysis
